# A Metabolomic Overview of Follicular Fluid in Cows

**DOI:** 10.3389/fvets.2018.00010

**Published:** 2018-02-08

**Authors:** Tatiane Melina Guerreiro, Roseli Fernandes Gonçalves, Carlos Fernando O. Rodrigues Melo, Diogo Noin de Oliveira, Estela de Oliveira Lima, Jose Antônio Visintin, Marcos Antônio de Achilles, Rodrigo Ramos Catharino

**Affiliations:** ^1^Innovare Biomarkers Laboratory, School of Pharmaceutical Sciences, University of Campinas – UNICAMP, Campinas, São Paulo, Brazil; ^2^Department of Animal Reproduction, College of Veterinary Medicine and Animal Science, São Paulo University – USP, São Paulo, Brazil; ^3^Achilles Genetics, Garça, Brazil

**Keywords:** follicular fluid, fertility, molecular markers, cows, oocyte quality

## Abstract

Follicular fluid (FF) protects the oocyte against proteolysis and extrusion during ovulation, providing an appropriate microenvironment that favors proper embryonic development; thereby, FF plays a key role in embryo quality. Being directly related to cattle breeding, studying FF is extremely important in livestock science to measure cattle fertility. This may eventually help to assess the quality of both meat and milk, products widely consumed worldwide. There is an important commercial interest in the evaluation and characterization of compounds present in the FF of livestock that present greater likelihood of pregnancy. Mass spectrometry is a great ally for this type of analysis and can provide quick and efficient screening for molecular markers in biological samples. The present study demonstrated the potential of high-resolution mass spectrometry in analyzing FF samples from two distinct groups of Nellore cows (*Bos indicus*): high and low fertility, as determined by the number of oocytes produced. We were able to delineate markers of interest for each group, which may ultimately be related to biochemical pathways that lead to higher or lower reproductive performance.

## Introduction

In the past 40 years, beef and dairy cattle were subject to intense selection, especially in terms of genetic engineering, primarily focusing on the improvement of milk and meat production (1). More recently, due to technological advances, it was observed that the infertility in dairy cows has decreased in the last few years (2, 3); nonetheless, despite optimistic numbers, infertility in cows is still a subject of great economic interest (4, 5). Hence, there is great demand to investigate the physiology and pathogenesis that are the triggering factors for infertility. It is currently known that this is a condition associated with both genetic and environmental elements, and the role of oocyte and embryo quality in the final fertility is the most noteworthy feature discussed in recent literature (6, 7). Newer platforms, such as transcriptomics, proteomics, and metabolomics, have been increasingly assisting researchers and clinicians in investigating and selecting oocytes and embryos using sophisticated methods. Follicular fluid (FF), follicular cells, and cumulus cells are fluids and tissues previously considered superfluous for analyses associated with oocyte quality. However, the trend of performing noninvasive sampling and evaluations has been increasing recently, giving a whole new relevance for the use of these samples (8).

Follicular fluid is responsible for providing oocyte protection against proteolysis, as well as aiding in the extrusion process during ovulation (9), acting as a buffer against adverse blood influences (10). Researchers have proposed and demonstrated in postpartum cows that there is a close correlation between the levels of certain metabolites in both: FF and blood serum. Thus, potential variations in serum concentrations of metabolites may affect FF, which may lead to changes in the quality of granulosa and oocyte cells (11). Therefore, since FF provides an appropriate microenvironment for the oocyte that leads to proper embryonic development, it is partly responsible for embryo quality (12, 13).

Currently, FF has been used for conservation and maturation of oocytes, with the purpose of performing *in vitro* fertilization in cattle (14). This use is justified due to the high protein content found in its constitution, even greater than that presented by fetal bovine serum, commonly used for this purpose (15). In addition, FF has been extensively explored in several works: biochemical constitution (13, 16, 17), factors that may influence or modify their production, such as climate (18) or nutrition (19), as well as its interference in the process of maturation of bovine embryo (14, 20).

Follicular fluid is formed through the transudation of the fluid produced by the theca and granulosa cells in the follicular antrum. This phenomenon occurs during the growth phase of the follicles, which increases the pressure inside the follicular antrum, expelling the fluid present there. Its composition and quantity can be modified during the development of the follicle (21, 22). Among its components, steroids and glycoproteins are found, which are synthesized by dominant follicular cells, and are part of the specific constituents of FF, as well as other factors or substances that are synthesized by ovarian somatic cells; these compounds contribute to the metabolism of cells and follicular oocyte (16, 23, 24). Also in the composition, there is a variety of polyunsaturated fatty acids, with linoleic acid found in larger quantities in small follicles. It is hypothesized that this may be one of the molecules responsible for inhibition of meiosis in bovine oocytes (25). More recently, studies related to estrogen activity with the composition of the fatty acids present in the FF of dairy cows, remaining different from the composition found in plasma (26).

Studying the composition of FF is of great importance, since it may be used as an evaluation parameter of oocyte quality, which can be directly related to fertility (27). In this way, FF analysis can be employed in livestock breeding strategies with the aim of improving milk and meat production. Commercial interest, therefore, implies the development of analytical techniques that provide fast and reliable results that are cost-effective at the same time to evaluate and characterize target compounds in FF (13, 27, 28). For this type of analysis, liquid chromatography techniques are commonly used, for example, LC-MS or HPLC-MS. For untargeted metabolomics screenings, however, LC-MS based systems are a considerably demanding alternative in terms of time and costs to identify unknown compounds in complex samples (29, 30). This is intimately related to the need of isolating compounds of interest first, and then performing characterization through comparison with certified standards (30), which makes it a much more suitable technique to be used in target analysis (31). In comparison to chromatography techniques, direct-infusion mass spectrometry (DIMS) with mass-selective detection is capable of providing high specificity chemical information, which includes molecular mass and/or characteristic information of the fragmented ions. This information can be used to identify compounds by matching the spectrum obtained with the data collected in databases of authentic compounds or to be used for *de novo* structural elucidation (31).

There is still little knowledge on the metabolomic profile of FF and, given the versatility of DIMS in providing fingerprints and selecting markers in biological samples (32–35), high-resolution mass spectrometry (HRMS) is ideal for fast screening, with minimal sample preparation and a high-throughput analytical process in shotgun lipidomic approach (36). The present study demonstrates the potential of HRMS to provide biomarkers for high and low fertility in cows (*Bos indicus*) from samples of FF, where it was possible to assign molecular markers to each group within a biochemical context, thereby demonstrating the sensitivity of this new methodology.

## Materials and Methods

### Animals

The Institutional Committee for Ethics in Animal Research of the University of Campinas (UNICAMP) is the body responsible for approving the handling of cattle used in this study. This academic institution follows the Ethical Principles of Animal Research, as established by the Brazilian College for Animal Experimentation (COBEA). Protocol number 2819-1 refers to this process and the research was executed strictly in accordance with the Public Health Service Policy.

The animals (*n* = 29) were bred in a tropical climate region (CwA Köppen classification), characterized by a rainy, hot summer, and a dry winter. The criteria used to select the animals in the study were: non-lactating mature Nellore (*Bos indicus*); age = 4–7 years old; body weight = 439 ± 20 kg. All cows were maintained on pasture (*Brachiaria decumbens* and *Brachiaria brizantha*) with mineral supplementation and water available *ad libitum*.

### Follicular Fluid

Samples of FF were obtained from all cows by follicular aspiration from ovaries with presence of corpora lutea and follicle diameter ranged 10–14 mm. FF was centrifuged at 1,000 x *g* for 1 min, and the supernatant was stored for analysis at −80°C. Samples were divided in three groups after FF aspiration, using the following the criteria: 12 animals that produced a higher number of oocytes (considered as *n* > 15), 9 animals that produced a lower number of oocytes <5, and 8 for control group, i.e., those producing an intermediate number of oocytes (considered as 5 < *n* < 15).

For analysis, samples were prepared by dilution (10:990 v/v) of FF in a solution of methanol and water (50:50 v/v). This first solution was filtered through 0.22-µm polyvinylidene difluoride membranes and resuspended (10:990 v/v) in a solution of methanol and water (50:50 v/v), resulting in a second solution. The second solution was divided in two vials (500 µL each) and either formic acid or ammonium hydroxide was added to a 0.1% concentration for analysis in positive and negative mode, respectively. All samples were prepared in triplicates. Methanol, ammonium hydroxide, and formic acid were purchased from J. T. Baker (Xalostoc, Mexico) and used with no further purification. Deionized water was obtained from a Milli-Q system (Millipore, USA).

### High-Resolution Mass Spectrometry

After preparation, all samples were injected for fingerprint analysis on an Orbitrap Discovery ESI-LTQ-XL instrument (Thermo Fisher Scientific, Bremen, Germany) with a nominal resolution of 30,000 (FWHM). Analyses were carried out in the mass range of 200–800 m/z. The instrument run was configured according to the following parameters: flow rate of 10 µL min^−1^, capillary temperature of 280°C, 5 kV of spray voltage, and sheath gas in 10 arbitrary units. HRMS acquisitions were performed in quintuplicates, and in both modes, positive and negative.

### Statistical Analysis and Biomarker Identification

The method of choice to evaluate the association between the groups was the partial least squares discriminant analysis (PLS-DA) with the variable importance in projection (VIP) score. This is a supervised method that uses multivariate regression techniques to extract features from each group and show the existence or not of differences and similarities between the analyzed samples. The statistical significance of the model obtained by PLS-DA was assessed by the application of two permutation tests: 10-fold cross validation and leave-one-out cross validation. Establishing a VIP score threshold greater than 3.0 was possible to perform the selection of characteristic biomarkers for each group. VIP score consists of the weighted average of squares of PLS loads and takes into account the amount of variance explained in each dimension used in the model. All chosen markers from the VIP scores list were submitted to receiver operating characteristics (ROC) curve analyses in order to verify the probability that each molecule had of belonging to their specific group. A heatmap of the markers elected was built using the Euclidean distance measurement and Ward clustering algorithms. All statistical analyses were performed using the online platform MetaboAnalyst 3.0 (37). For the structural elucidation of the markers, mass accuracy was the main parameter, by comparing the mass values obtained experimentally and those available in online databases, such as METLIN (Scripps Center for Metabolomics, La Jolla, CA, USA), in order to guide the choice of potential markers for the quality of bovine oocytes. A molecule was deemed characterized when presented an identification error value of less than 2 ppm.

## Results

**Figure 1:**
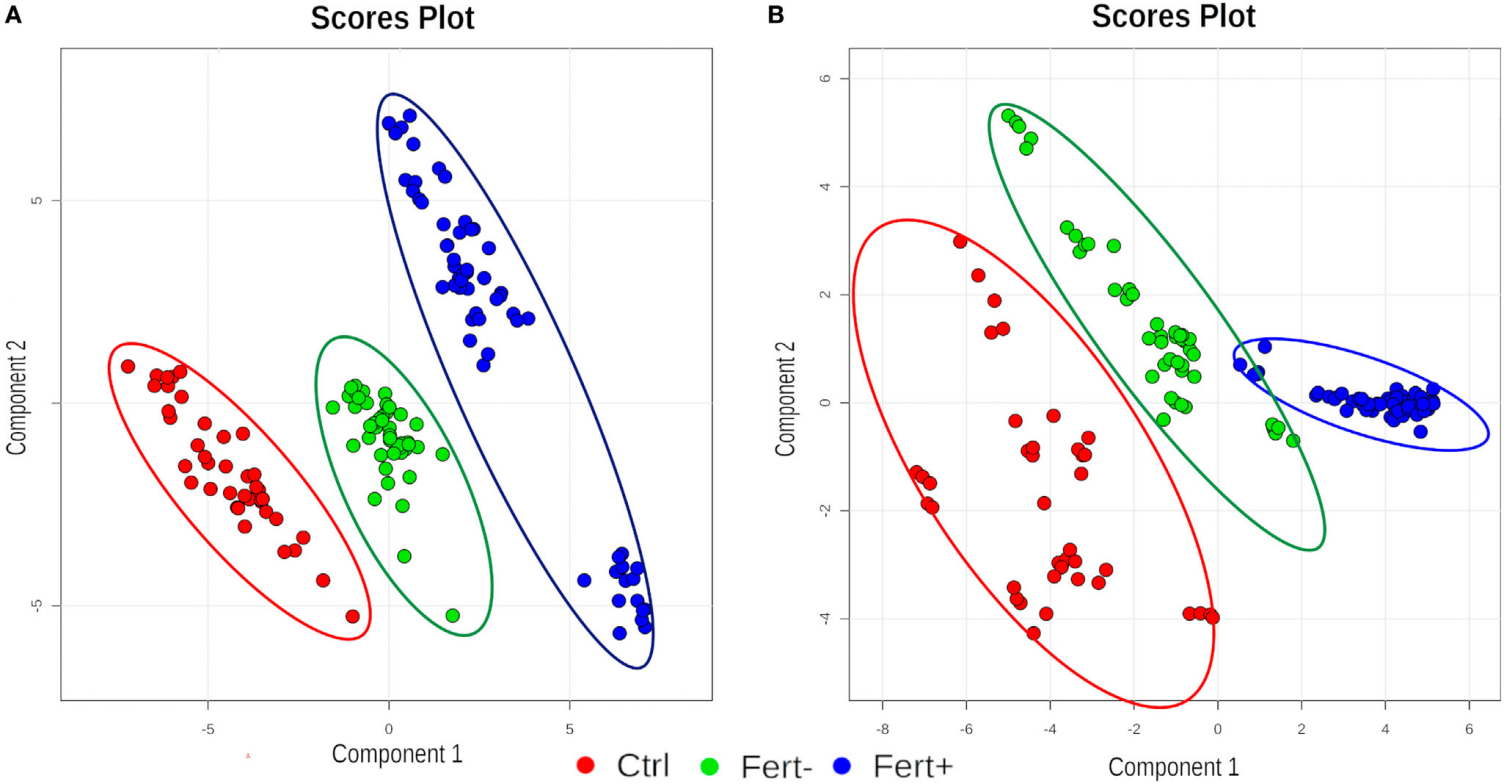
Clustering graphs of partial least squares discriminant analysis analysis for all groups (Ctrl, control; Fert+, higher number of oocytes; Fert−, lower number of oocytes) at positive **(A)** and negative **(B)** ion mode.

Figure 1 shows the clustering graphs of PLS-DA analysis, where it was possible to assess separation among all groups, in both ion modes: positive (Figure 1A) and negative (Figure 1B). Statistical and chemical analyses combined provided all markers, as follows: 7 candidates for the group with a higher number of oocytes, split into three markers for the positive ion mode and four for the negative ion mode, and two candidate markers selected for the group with a lower number of oocytes, split as one in the negative and one in the positive ion mode. The compounds are organized in Table 1.

**Table 1:** Biomarkers of the groups of high and low number of oocytes in the positive and negative modes of analysis.

	Experimental mass	Theoretical mass	Error (ppm)	Adduct	Compound	MID*
Higher number of oocytes	429.0954	429.0946	1.8644	[M + K]^+^	Resveratrol 4′-glucoside	87064
	455.1715	455.1706	1.9773	[M + H-H_2_O]^+^	Lupinisoflavone N	47791
	667.1881	667.1869	1.7986	[M + H]^+^	Peonidin acetyl 3,5-diglucoside	47015
	243.0659	243.0663	1.6539	[M-H]^−^	3,3′,4,5′-Tetrahydroxy-trans-stilbene	7029
	319.1328	319.1334	1.9114	[M-H_2_O-H]^−^	5,7-dihydroxy-6-methyl-8-prenylflavanone	52673
	335.1277	335.1283	1.8202	[M-H]^−^	Xanthohumol	52097
	363.1223	363.1216	1.9277	[M + Cl]^−^	Prostaglandin M	45949

Lower number of oocytes	476.3168	476.3159	1.8895	[M + H]^+^	*N*-docosahexaenoyl phenylalanine	75476
	336.3260	336.3266	1.8137	[M-H_2_O-H]^−^	*N*-Eicosanoyl-ethanolamine	3724

*METLIN ID.

**Figure 2:**
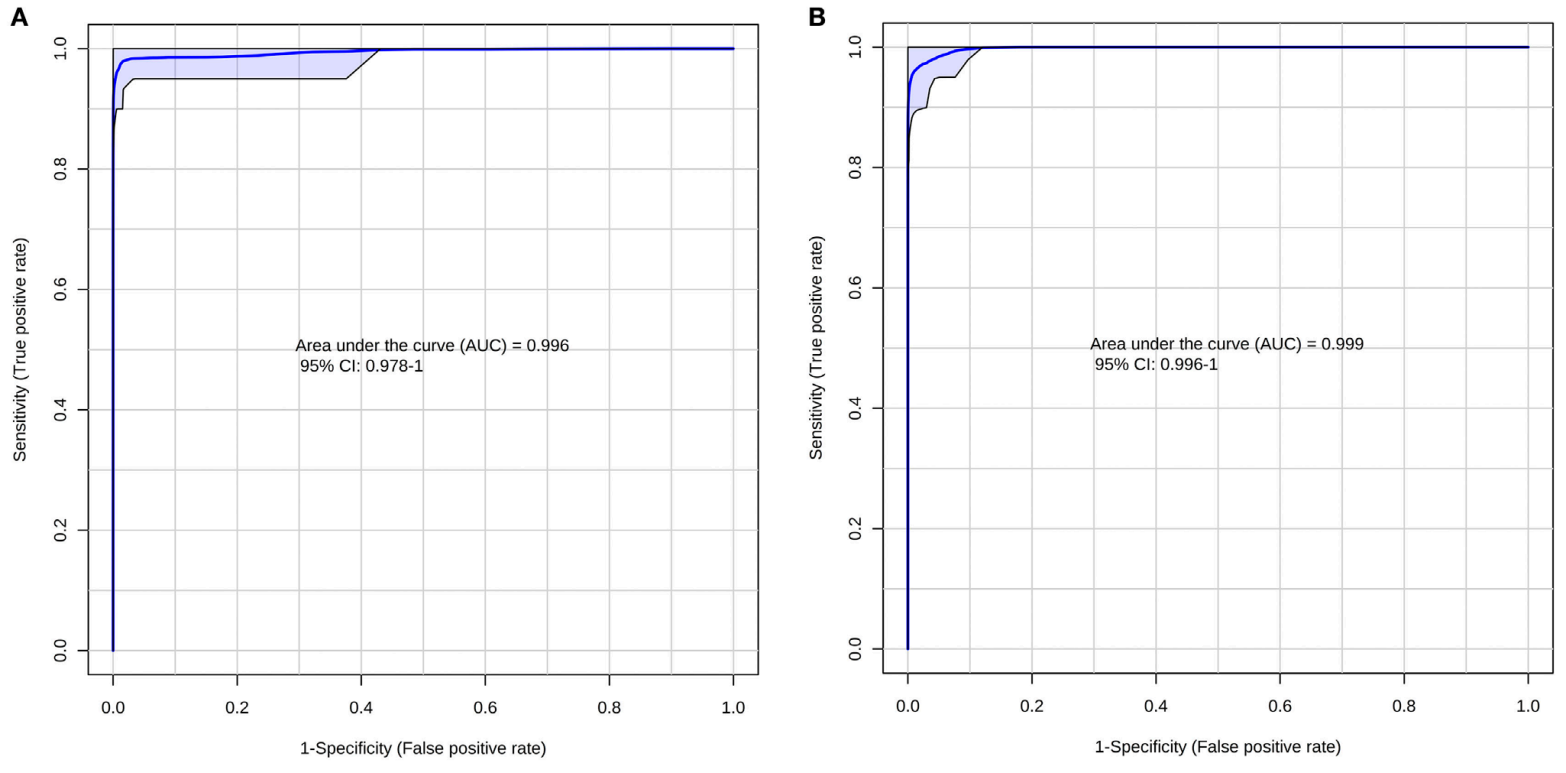
Receiver operating characteristics curve for the group with higher number of oocytes at positive **(A)** and negative **(B)** ion mode.

**Figure 3:**
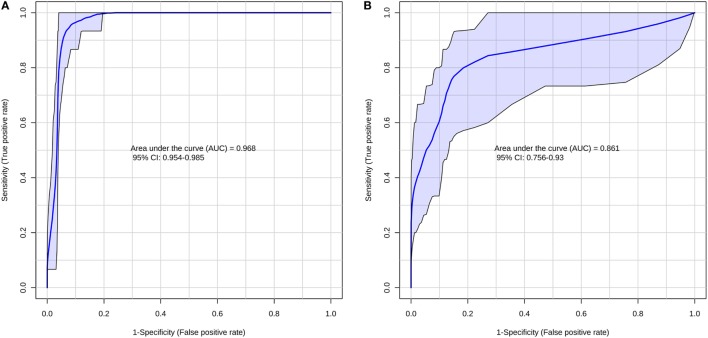
Receiver operating characteristics curve for the group with lower number of oocytes at positive **(A)** and negative **(B)** ion mode.

**Figure 4:**
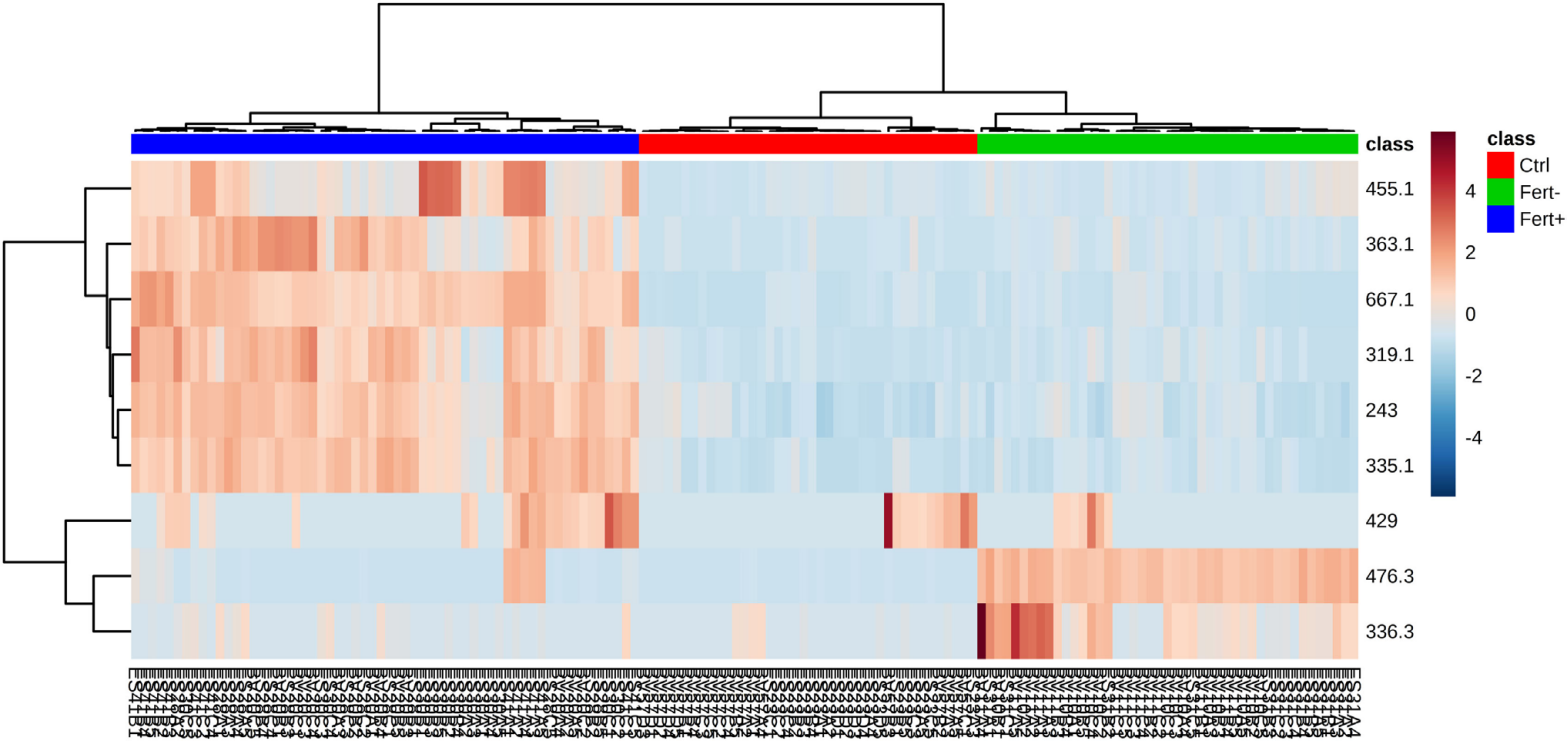
Heatmap (distance measured by Euclidean and Ward clustering algorithms), with a color-coded thermometer (right) indicating the expression of each biomarker on each respective group. Fert+, higher number of oocytes; Fert−, lower number of oocytes; Ctrl, control.

According to the ROC curve analysis, the group with higher number of oocytes achieved satisfactory results on both negative and positive ion modes. The three markers for the high number of oocytes group in the positive ion mode presented an area under the curve (AUC) value of 0.996 (0.978–1.00), with a sensitivity and specificity values of 0.950 (0.851–0.987) and 0.992 (0.950–1.000), respectively (Figure 2A). In addition, the four markers obtained in the negative ion mode for the same group achieved an AUC of 0.999 (0.996–1.000) with sensitivity and specificity values of 0.932 (0.827–0.978) and 1.000 (0.961–1.000), respectively (Figure 2B). A similar behavior was observed with the markers selected for the group with lower number of oocytes; the marker selected from the positive ion mode presented an AUC of 0.968 (0.954–0.985) with values of sensitivity and specificity of 0.978 (0.868–0.999) and 0.864 (0.794–0.914), respectively (Figure 3A). Finally, the marker from the negative ion mode in the same group was achieved an AUC of 0.861 (0.756–0.930) with values of sensitivity and specificity of 0.733 (0.578–0.849) and 0.887 (0.817–0.933), respectively (Figure 3B). The selected and characterized markers are presented in the Heatmap (Figure 4), showing the presence or absence of these markers among the groups.

## Discussion

Results indicated a remarkable possibility of employing FF analysis as a reliable test to predict whether a particular animal will present a higher or lower number of oocyte production rate. This information is important, as the number of oocytes was directly related to the increased fertility in cows; hence, the higher the number, the greater will be the chance of reproduction (38). From the results in Table 1, it is possible to infer two main differences: in the group of animals with low oocyte number, the statistical model elected compounds that are directly related to an inflammatory state that, consequently, increases oxidative stress and, even more interestingly, may also be involved with negative hormonal feedback. On the other hand, for the high oocyte number group, compounds were mostly phenolics and flavonoids, i.e., species that are probably incorporated from feed, given their non-endogenous character, and are closely related to protection against oxidative stress in the embryo-developing environment.

In the low oocyte production group, *N*-docosahexaenoyl phenylalanine was elucidated in the positive mode, and *N*-eicosanoyl ethanolamine in the negative mode. *N*-docosahexaenoyl phenylalanine is part of the group of *N*-acyl amides and *N*-eicosanoyl ethanolamine is part of the group of *N*-acyl ethanolamines. These compounds constitute a general class of endogenous simple fatty amides, characterized by an acyl group bonded to a nitrogenated moiety, i.e., a conjugated system that comprises putative signaling molecules with a wide range of biological activity (39). The best-known subgroup of this classification would be the endocannabinoids, which are endogenously produced lipids capable of binding to cannabinoid receptors. In addition, other types of endogenous lipids, similar to endocannabinoids in structure, are not capable to bind with cannabinoid receptors. Despite that, they demonstrate cannabimimetic effects, similar to the markers found in this work (39).

Cannabinoid receptors 1 and 2 (CB1 and CB2) have been described in human endometrium (40), where CB1 signalizes for the transport of embryos through the oviduct (41) as well as for embryo establishment in the womb (42). Recent contributions have shown that the endocannabinoid anandamide plays an important role during the fertilization process in humans (43), as well as the influence of anandamide in the estrous cycle in bovines has been also reported (44). Given that the ovulation process is quite similar in mammalians, it was shown in both studies that high concentrations of anandamide are related to the hormonal peak of estradiol during ovulation, responsible for the release of the ovule. In addition, it has been reported that high levels of anandamide in humans are related to early pregnancy loss (45) and reduction in levels of progesterone (40), a key hormone for both implantation of the ovum and maintenance of pregnancy (43, 46, 47). As *N*-docosahexaenoyl phenylalanine has cannabimimetic effects, its presence in the FF of cows from the lower oocyte number group could be related to this hormonal regulation presented by anandamide. In the case of *N*-eicosanoyl ethanolamine, its presence might be related to the “entourage effect,” in which these *N*-acyl ethanolamines inhibit anandamide degradation through their ability to compete for fatty acid amide hydrolase, since both compounds are hydrolyzed by the same enzymatic reaction. With increasing levels of anandamide, progesterone remains low; this condition does not allow the occurrence of positive pregnancy, as the high concentration of progesterone in FF is related to the increase of fertility in cattle (46, 47).

Moreover, eicosanoids and docosanoids are extensively described in literature as key intermediates in the inflammation cascade, and their presence is often regarded as associated with an existing condition that may be associated to an inflammatory process (48). The influence of inflammation and the underlying oxidative environment caused by this process are also widely discussed regarding their negative influence over reproduction (49). Therefore, the election of *N*-docosahexaenoyl phenylalanine and *N*-eicosanoyl ethanolamine as markers for the low oocyte number group also make sense considering the potential oxidative stress existing in animals from these groups.

Regarding the high oocyte number group, by observing Table 1, we find Resveratrol 4′-glucoside, Lupinisoflavone N, and Peonidin acetyl 3,5-diglucoside in the positive ion mode; on the other hand, in the negative mode, we find 3,3′,4,5′-tetrahydroxy-trans-stilbene, 5,7-dihydroxy-6-methyl-8-prenylflavanone, xanthohumol, and prostaglandin M. The main characteristic that is common to great majority of these species is the antioxidant character associated with these compounds, as oocytes and embryos are highly vulnerable to oxidative stress and other conditions when cultured *in vitro* (50).

Resveratrol 4′-glucoside and 3,3′,4,5′-tetrahydroxy-trans-stilbene, or piceatannol, are compounds that belong to the class of stilbenes. Piceatannol is a metabolite of resveratrol that has greater antioxidant potential than its precursor due to the position of its hydroxyl groups, which favors the capture of free radicals (51). Some contributions in the literature have discussed the benefits of resveratrol during mammalian reproduction, including the improvement on the quality of bovine embryos when added during oocyte maturation, making these embryos more resistant during cryopreservation (52). In addition, when resveratrol was consumed for a longer period, it improved and increased the number of oocytes produced by female mice (52). Since the cows in this study were fed green pasture, it is plausible to have Resveratrol 4′-glucoside elected as a marker for the group of cows with high oocyte number production, as its high antioxidant potential (53) favors both the oocyte maturation process and the future development of new embryos in cattle.

The other elected markers may also be attributed to come from the diet and belong to the family of flavonoids, which are compounds characterized by the presence of interconnected phenolic rings, directly related to the antioxidant potential presented by its members (54). This family has several subclassifications of its components, according to specific characteristics of the chemical structure they present, being able to comprise isoflavones, anthocyanidins, and prenylflavonoids, which have, respectively, the markers Lupinisoflavone N, peonidin acetyl 3,5-diglucoside, 5,7-dihydroxy-6-methyl-8-prenylflavanone, and xanthohumol. Lupinisoflavone N, which is an isoflavone found in plants of the genus Lupine, from the family Leguminosae (55). Isoflavones are phenolic compounds and have a well-known antioxidant character (56, 57); studies have shown that supplementation of animal feeding with lupines is related to increased ovulation rate in sheep (58) and also to improved reproductive efficiency in the case of ruminants (59). Peonidin acetyl 3,5-diglucoside belongs to the class of anthocyanins, a class of flavonoids with high antioxidant potential (60–62). With respect to these characteristic, we have another marker that could be collaborating for the development of embryos due to its ability to protect oocytes from free radicals or reactive oxygen species (ROS) (62).

Regarding the subclass of prenylflavonoids, we have 5,7-dihydroxy-6-methyl-8-prenylflavanone and xanthohumol. This class of bioactive compounds are prenylated phenylalanine derivatives formed in the plant secondary metabolism and may be found in several species. The activity of this class relies on phytoestrogenic and antioxidant properties (63), and studies have shown that xanthohumol has anticancer, antidiabetic, antibacterial, and anti-inflammatory activities (64, 65). These characteristics may be related to its antioxidant capacity, since xanthohumol can act directly reducing the formation of ROS, or indirectly through the induction of cellular defense mechanisms against oxidative stress, thus contributing to the improvement of several diseases related to ROS (65–68). The presence of compounds such as xanthohumol in the FF may be traced back directly to the animal’s feed, since flavonoids and its derivatives are ubiquitous to the metabolism of several plant species, especially grasses such as *B. decumbens* and *B. brizantha* (69, 70), which were used in the pastures of the animals in this study.

Finally, the last marker found in the negative mode for the high fertility group was Prostaglandin M. Prostaglandins (PGE) are a group of physiologically active lipids due to their ability to generate hormonal responses in animals. PGE-M is a metabolite of PGE-2 (71), which in turn is directly linked to ovulation as it increases its levels when there is an increase in luteinizing hormone (72). PGE-2 is one of several signaling molecules that, together with the follicle, are able to coordinate oocyte maturation, and enhanced expression of proteases associated with follicle rupture, which ensures the release of an optimally mature oocyte during ovulation (73–75). Hence, the presence of PGE-M as a marker of the high oocyte number group is indicative of a higher performance of PGE-2 in the animals of this group.

Through our findings, it was possible to establish a link between markers found in bovine FF and oocyte quality/production rate. Incidentally, we expected that the relevant compounds were simply derived from feed and that are also potentially related to the increase of fertility in cows, since they were the chosen markers for the group of animals with higher production of oocytes. As we observed, however, the inflammatory status of the animals is what indeed plays a key role in the oocyte production and, ultimately survival; docosanoids and eicosanoids, the molecular classes observed in the low oocyte group, are well-known players involved in reactions that lead to inflammatory state in organisms. This provides evidence that, even though the feed is the same for both animal groups, there is an important depletion of antioxidants in the low oocyte group due to a potentially high oxidative stress to which these animals are subjected. Hence, antioxidants are in high levels for the high-oocyte animals and, therefore, were elected as markers, as opposed to the low-oocyte, which present an inflammatory picture, traced back from the molecular classes elected by PLS-DA.

## Ethics Statement

The Institutional Committee for Ethics in Animal Research of the University of Campinas (UNICAMP) is the body responsible for approving the handling of cattle used in this study. This academic institution follows the Ethical Principles of Animal Research, as established by the Brazilian College for Animal Experimentation (COBEA). Protocol number 2819-1 refers to this process and the research was executed strictly in accordance with the Public Health Service Policy.

## Author Contributions

RG, JV, and MA performed sample collection. TG performed experiments and wrote the manuscript. TG and CM performed data analysis. DO and EL performed manuscript review. RC idealized all experiments and managed the research group.

## Conflict of Interest Statement

The author MA is employed by company Achilles Genetics. All other authors declare no competing interests of any kind.
